# miR-132 serves as a diagnostic biomarker in gestational diabetes mellitus and its regulatory effect on trophoblast cell viability

**DOI:** 10.1186/s13000-019-0899-9

**Published:** 2019-10-25

**Authors:** Xuegui Zhou, Cuiping Xiang, Xiaoxia Zheng

**Affiliations:** 1 Department of Obstetrics, Binzhou People’s Hospital, No. 515, Huanghe 7 Road, Binzhou, Shandong 256610 People’s Republic of China; 2 Department of Obstetrics, Binzhou Center Hospital, Binzhou, Shandong 251700 People’s Republic of China

**Keywords:** Gestational diabetes mellitus, MicroRNA-132, Trophoblast cell, Proliferation, Diagnosis

## Abstract

**Background:**

Gestational diabetes mellitus (GDM) leads to poor pregnancy outcomes. Strategies that improve trophoblast cell function are important methods for GDM treatment. This study aimed to investigate the expression and diagnostic potential of microRNA-132 (miR-132) in GDM patients, and further analyzed the effects of miR-132 on HTR-8/SVneo cell proliferation.

**Methods:**

Quantitative real-time PCR was applied to estimate the expression of miR-132. A receiver operating characteristics curve (ROC) analysis was performed to evaluate the diagnostic value of serum miR-132 in GDM patients. In vitro regulation of miR-132 in trophoblast cell HTR-8/SVneo was achieved by cell transfection, and the effects of miR-132 on cell proliferation were assessed using CCK-8 assay.

**Results:**

Expression of miR-132 was decreased in serum and placenta tissues in GDM patients compared with the healthy women. A negative correlation was found between the serum miR-132 levels and fasting blood glucose of the GDM patients. A ROC curve shown the serum miR-132 had considerable diagnostic accuracy with an area under the curve (AUC) of 0.898. High glucose (HG) treatment induced an inhibition in HTR-8/SVneo cell proliferation and the expression of miR-132. The overexpression of miR-132 in HTR-8/SVneo cells could markedly rescued the HG - induced suppressed cell proliferation.

**Conclusion:**

All the data of this study revealed the reduced expression of miR-132 in serum and placenta tissues of GDM, and serum miR-132 serves a candidate biomarker in the diagnosis of GDM. miR-132 may act a protective role against GDM via enhancing the trophoblast cell proliferation.

## Introduction

Gestational diabetes mellitus (GDM) is a special kind of diabetes mellitus, which first occurs in pregnancy with impaired glucose tolerance [1]. Statistical data reveal that there is approximately 18.9% of pregnant women diagnosed with GDM in China [2]. GDM induces numerous complications and leads to poor pregnancy outcomes, including abortion, fetal malformation, fetal growth restriction, premature rupture of membrane and macrosomia [3]. Similar to general diabetes mellitus, hyperglycemia and insulin resistance are also the characteristics of GDM patients [4]. Thus, GDM has great potential to progress to type 2 diabetes mellitus (T2DM) if no any lifestyle, dietary or pharmacological intervention are administrated [5]. Thus, early diagnosis is very important for the women with GDM.

High glucose (HG) as one of the major characteristics of GDM has been reported to impair the normal development of placenta, resulting in the increases in abortion and fetus malformation [6]. Placenta is a pivotal organ for nutrition exchange between fetus and mother, its regular growth and development are premises and guarantees of a healthy pregnancy [7]. During the development placenta, normal function of trophoblast cells is one of the most critical events. The impairment in trophoblast cell proliferation can lead to the maldevelopment of placenta tissues [8, 9]. However, the understanding about the mechanisms underlying the regulation of trophoblast cell biological function in GDM remains limited and urgently needs to be uncovered.

MicroRNAs (miRNAs) are a class of small noncoding RNAs and involved in numerous disease progressions, including the development of GDM [10, 11]. The dysregulation of miRNA has dramatic clinical significance in diagnosis and prognosis of human diseases, and can be used as efficient therapeutic targets due to their critical regulatory roles in various cellular processes, such as proliferation, migration, invasion and apoptosis [12–15]. MicroRNA-132 (miR-132) has been previously reported to be downregulated in GDM patients [16], but its clinical and biological function in GDM are rarely reported.

This study firstly investigated the expression of miR-132 in serum and placenta tissues of GDM, and further evaluated its diagnostic potential for GDM patients. By using trophoblast cell HTR-8/SVneo, this study sought to uncover the functional role of miR-132 in the regulation of cell proliferation.

## Materials and methods

### Patients and sample collection

One hundred and eight GDM patients and 50 healthy pregnant women as a control group were enrolled in this study from Binzhou People’s Hospital between 2013 and 2017. The diagnosis of GDM was determined following the guidelines of American Diabetes Association [17]. The cases with pre-gestational diabetes, multiple gestation accompanied with further complications and had medication were excluded from this study. Blood samples were collected from the participants at the 24–28 pregnancy weeks after an overnight fast, and fasting blood glucose was measured and recorded. Placental tissues were collected from the participants at the time of delivery, then stored in liquid nitrogen for further examinations. All the participants signed the informed consent for sample acquisition for research purposes. The protocols of this study were approved by the Ethics Committee of Binzhou People’s Hospital.

### Cell culture and treatment

Human trophoblast cell lines HRT-8/SVneo and BeWo was obtained from the Chinese Academy of Sciences Cell Bank (Shanghai, China). HRT-8/SVneo was cultured in Dulbecco’s modified Eagle’s medium (DMEM; Invitrogen, MA, USA) supplemented with 1000 mg/L glucose and 10% fetal bovine serum (FBS; Gibco, CA, USA), and BeWo was cultured in cultured in F-12 medium (Gibco, CA, USA) added with 10% FBS at 37 °C in a humidified atmosphere containing 5% CO_2_. The cells were divided into two groups, including HG treated group and normal cell group. Cells in the HG group were cultured in a HG medium with the glucose concentration of 25 mM, while the cells in the normal group were incubated in the normal culture medium (glucose concentration of 5 mM).

### Cell transfection

Cell transfection vectors, including miR-132 mimic, miR-132 inhibitor and the separate negative control (mimic NC and inhibitor NC), were purchased from GenePharma (Shanghai, China). These vectors were respectively transfected into the trophoblast cells using Lipofectamine 3000 (Invitrogen, Carlsbad, CA, USA) following the manufactures’ instructions. After 48 h of the transfection, the cells were used for the next experiments.

### RNA extraction and quantitative real-time PCR (qRT-PCR)

Total RNA was extracted using the TRIzol reagent (Invitrogen, Carlsbad, CA, USA) and its quality was estimated using a NanoDrop 2000 (Thermo Fisher Scientific, Waltham, MA, USA). cDNA was synthesized from the RNA according to a PrimeScript RT reagent kit (TaKaRa, Shiga, Japan) as per the protocols of manufactures. The expression of miR-132 was examined using qRT-PCR, which was carried out using a 7300 Real-Time PCR System (Applied Biosystems, USA) and a SYBR green I Master Mix kit (Invitrogen, Carlsbad, CA, USA). The final expression values were calculated using the 2^−ΔΔCt^ method and normalized to U6.

### Cell proliferation assay

After cell transfection, the trophoblast cells were seeded into 96-well culture plates for the cell proliferation examination using a Cell Counting Kit-8 (CCK-8; Beyotime, Shanghai, China). The cells were cultured at 37 °C for 3 days, and the CCK-8 reagent was added into the wells every 24 h following with a 4 h further incubation. The cell proliferation was evaluated by reading the absorbance of cell cultures at 450 nm using a microplate reader (Molecular Devises, CA, USA).

### Statistical analysis

Data in this study were presented as mean ± SD and analyzed using SPSS 21.0 software (SPSS Inc., Chicago, IL) and GraphPad Prism 7.0 software (GraphPad Software, Inc., USA). Comparisons between groups were assessed using Student’s t test or one-way ANOVA. Pearson correlation analysis was performed to estimate the correlations between parameters. A receiver operating characteristics curve (ROC) was plotted to evaluate the diagnostic value of miR-132 in the patients with GDM. A result with *P* < 0.05 was considered statistically significant.

## Results

### Baseline characteristics of the participants

A total of 158 pregnant women were enrolled in our study, including 50 healthy controls and 108 GDM patients. The demographic and clinical features of the participants were compared and no significant difference was observed in age, BMI and pregnancy weeks between the two groups (all *P* > 0.05, Table 1). Compared with the healthy women, the fasting blood glucose was markedly higher in the women with GDM (*P* < 0.001).

**Table 1:** Demographic and clinical characteristics of the women included in this study

Parameters	Healthy controls (*n* = 50)	GDM patients (*n* = 108)	*P* value
Age (years, mean ± SD)	30.10 ± 2.79	30.89 ± 3.45	0.159
BMI (kg/m^2^, mean ± SD)	20.10 ± 1.65	22.82 ± 2.57	0.064
Pregnancy weeks (weeks, mean ± SD)	25.32 ± 1.45	25.20 ± 1.23	0.602
Fasting blood glucose (mM/L, mean ± SD)	4.43 ± 0.27	6.31 ± 0.31	< 0.001

*GDM* gestational diabetes mellitus; BMI, body mass index

### Expression of miR-132 in serum and placenta tissues of GDM

**Fig. 1:**
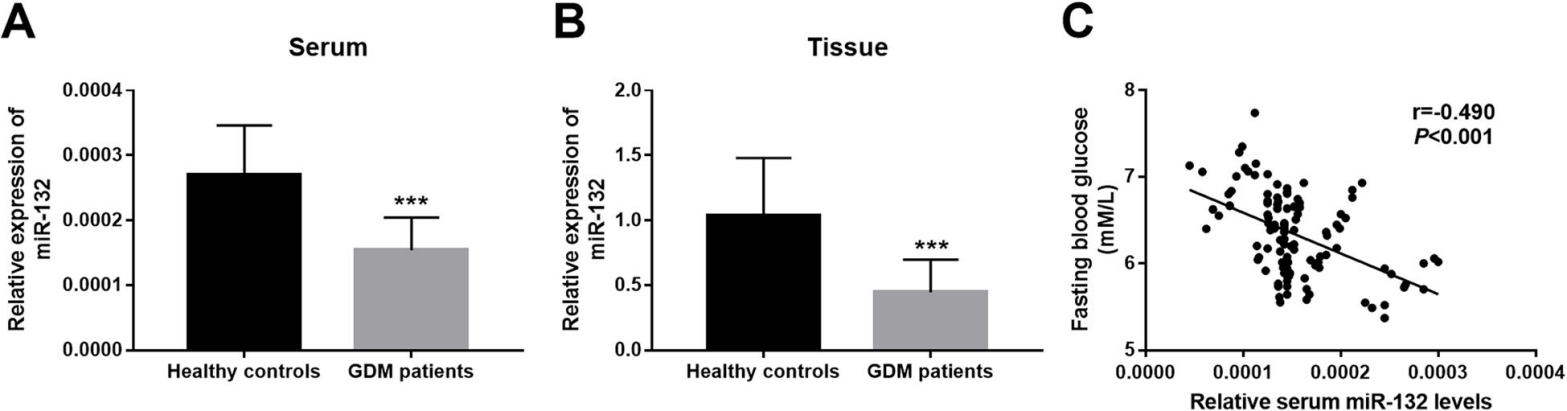
Expression of miR-132 and its correlation with fasting blood glucose in patients with GDM. **a** and **b**. Expression of miR-132 in serum (**a**) and placenta tissue (**b**) was downregulated in GDM patients compared with the healthy women. **c**. Serum miR-132 levels were negatively correlated with the fasting blood glucose of the GDM patients (r = − 0.490, *P* < 0.001). ***P < 0.001

As shown in Fig. 1a, we observed a significant decrease in serum expression of miR-132 in the GDM patients compared with the healthy controls (*P* < 0.001). Placental tissue of the participants during delivery were collected to further examine the expression patterns of miR-132. Similar to the serum data, the expression of miR-132 in the placental tissues was also obviously decreased in the GDM patients in a comparison to the healthy controls (*P* < 0.001, Fig. 1b). Increased blood glucose is a characteristic of GDM, and the fasting blood glucose was as expected elevated in the GDM patients in this study. This study further found a negative correlation of the serum miR-132 expression with the patients’ fasting blood glucose levels (*r* = − 0.490, *P* < 0.001, Fig. 1c).

### Diagnostic value of miR-132 for patients with GDM

**Fig. 2:**
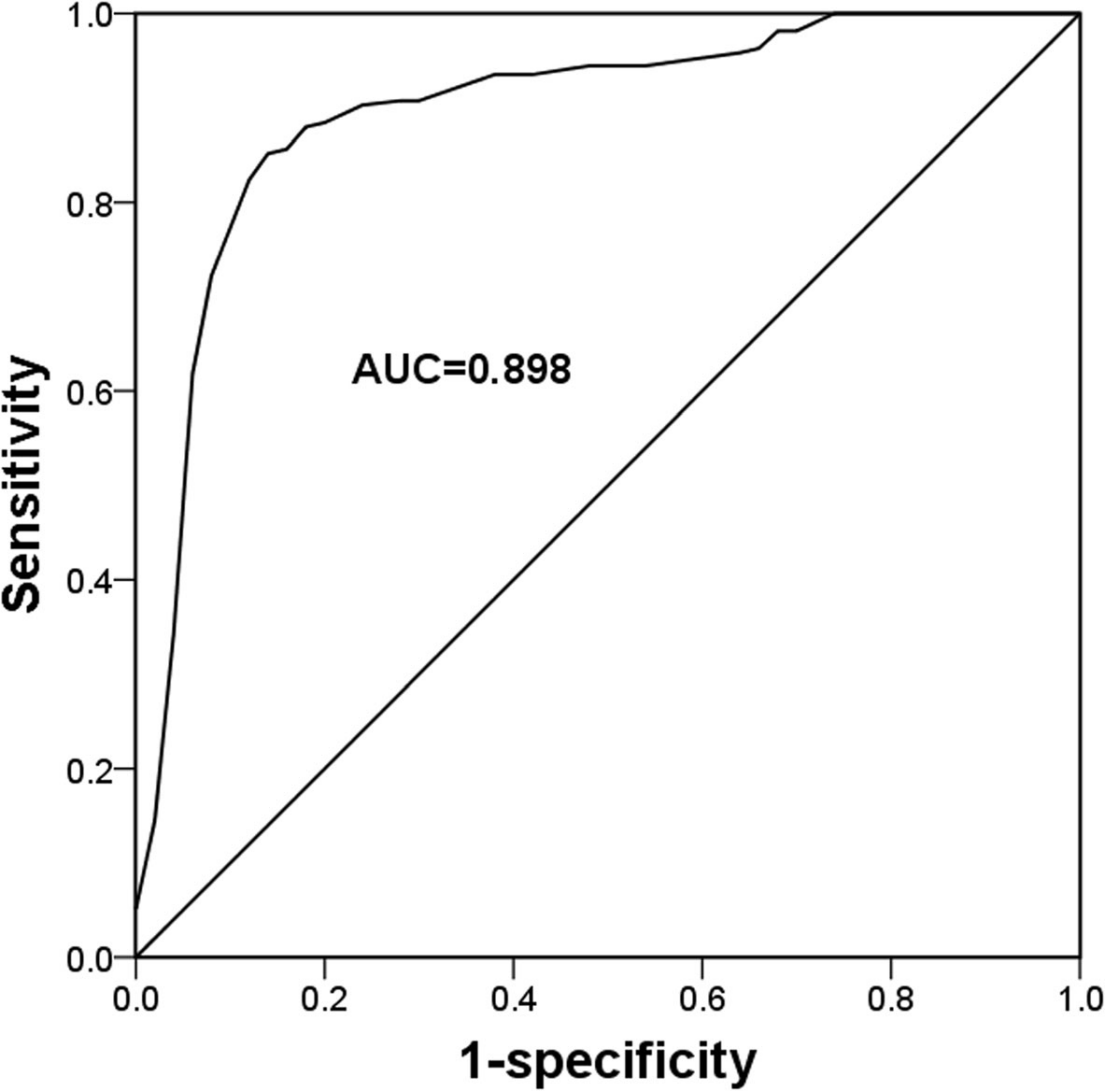
A ROC curve based on serum miR-132 in GDM patients. The area under the curve (AUC) was 0.898 for serum miR-132 expression to distinguish GDM cases from healthy pregnant women

Since the serum expression of miR-132 in GDM patients was remarkably different from the healthy controls, the diagnostic potential of miR-132 was further investigated. A ROC curve (Fig. 2) constructed based on serum miR-132 levels revealed that the area under the curve (AUC) was 0.898, indicating the relative high diagnostic accuracy of miR-132 for the differentiation between GDM patients and healthy women. The diagnostic sensitivity and specificity were respectively 84.3 and 88.0% at a cutoff value of 1.995 × 10^− 4^.

### HG suppresses trophoblast cell viability and the expression of miR-132

**Fig. 3:**
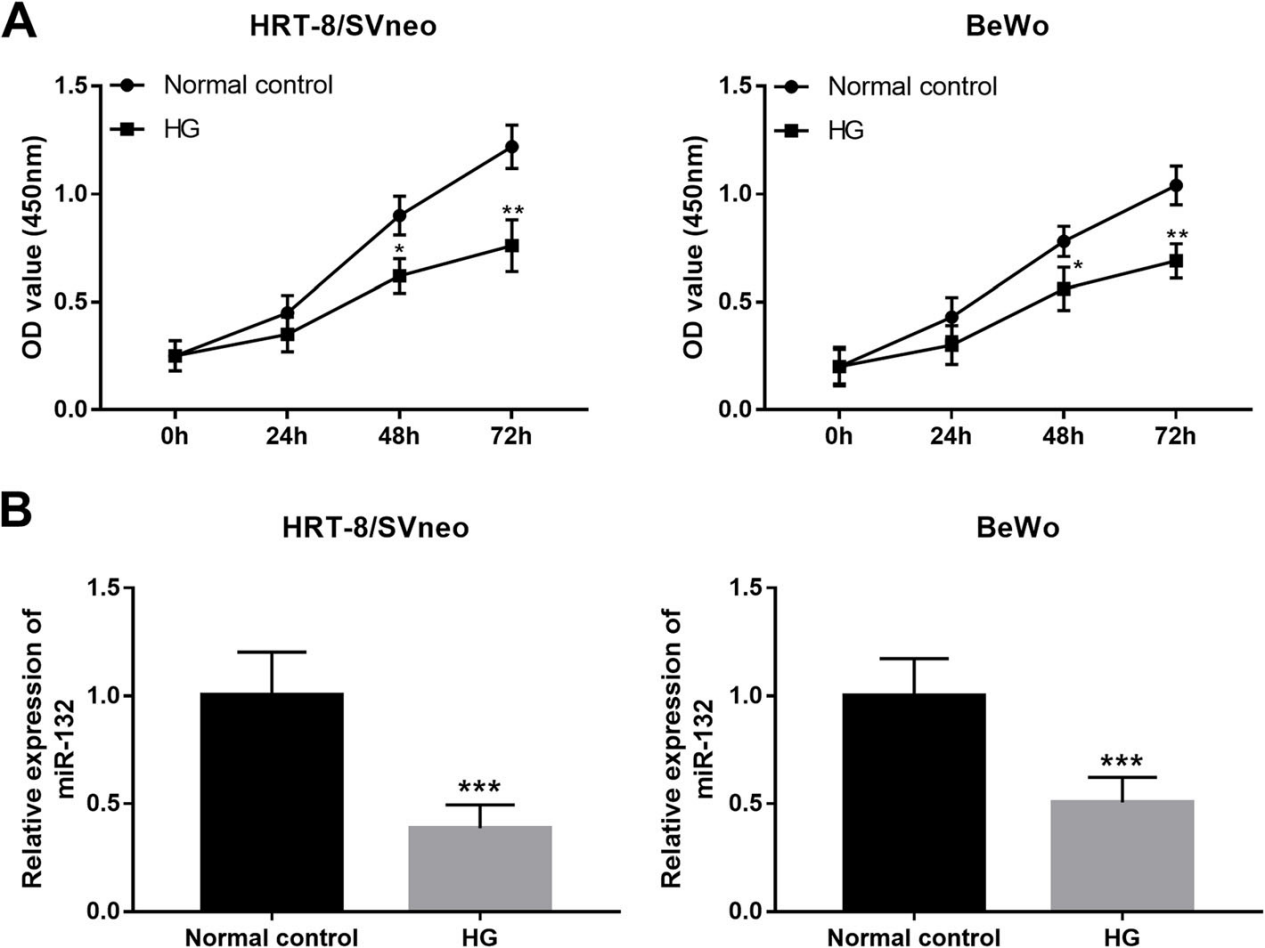
HG suppressed cell proliferation (**a**) and inhibited the expression of miR-132 (**b**) in two trophoblast cell lines HTR-8/SVneo and BeWo. HG, high glucose; *P < 0.05, **P < 0.01, ***P < 0.001

HG in GDM patients leads to inhibited trophoblast cell proliferation, thus resulting in the impaired placental development. From Fig. 3a, the cell proliferation of trophoblast cells cultured in HG medium was as expected significantly suppressed compared with the normal controls (*P* < 0.05). Similar to the serum expression data in GDM patients, the expression of miR-132 was also reduced by HG in trophoblast cells when compared to the untreated cells (*P* < 0.001, Fig. 3b).

### Overexpression of miR-132 contributes trophoblast cell viability upon HG treatment

**Fig. 4:**
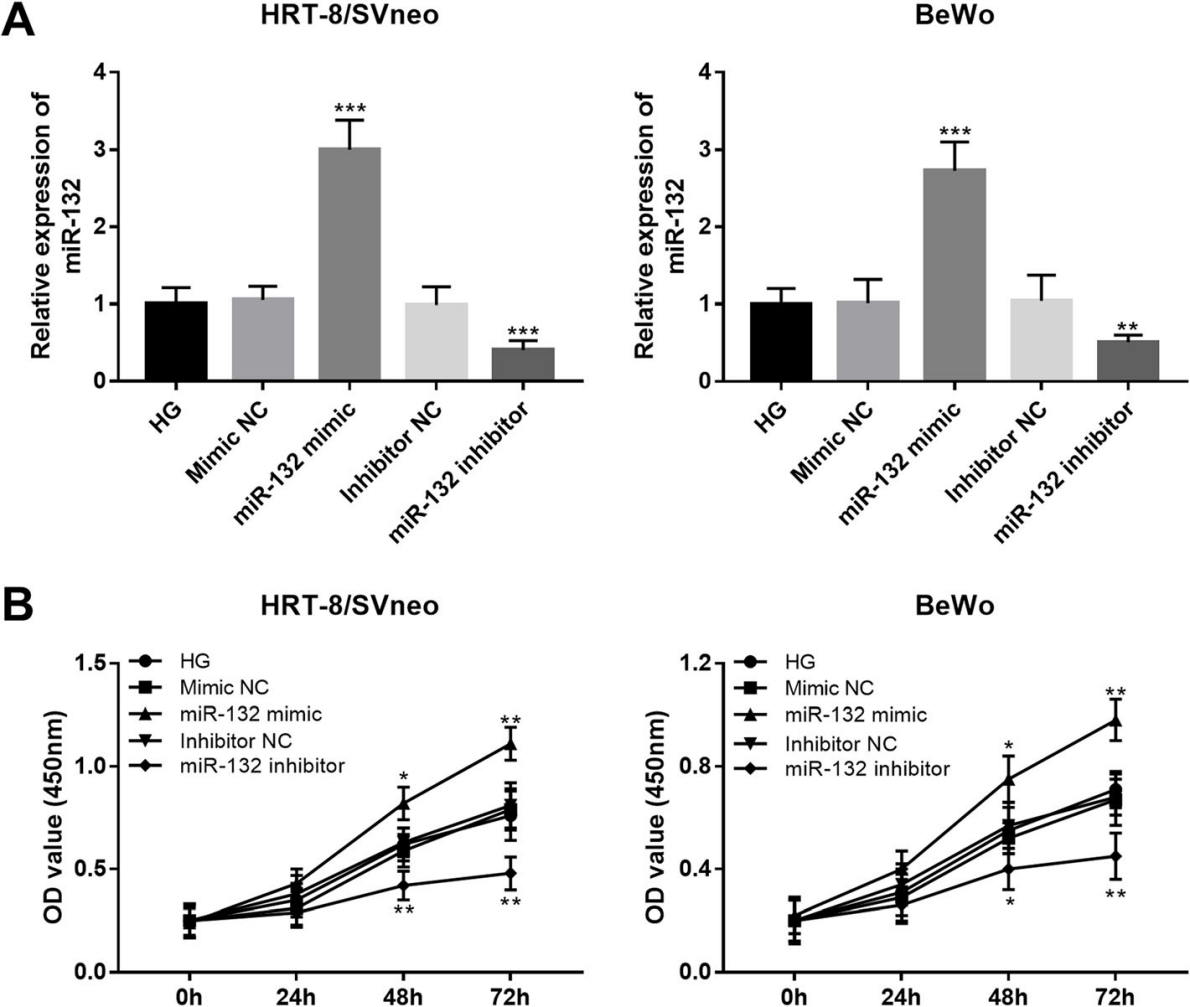
Effects of miR-132 on cell proliferation of trophoblast cells upon HG treatment. **a**. Expression of miR-132 was upregulated by the miR-132 mimic, but was downregulated by the miR-132 inhibitor in both HTR-8/SVneo and BeWo cell lines. **b**. The upregulation of miR-132 promoted cell proliferation, while the downregulation of miR-132 inhibited cell proliferation in HTR-8/SVneo and BeWo cells. HG, high glucose; *P < 0.05, **P < 0.01, ***P < 0.001

Since the expression of miR-132 was inhibited in trophoblast cells upon HG treatment, this study sought to investigate whether the regulation of miR-132 could lead to any change of cell viability of the trophoblast cells. By cell transfection, the expression of miR-132 was upregulated by the miR-132 mimic, but was downregulated by the miR-132 inhibitor in trophoblast cells (all *P* < 0.001, Fig. 4a). According to CCK-8 assay, we found that the overexpression of miR-132 could promote the trophoblast cell proliferation, while the knockdown of miR-132 resulted in the inhibited cell proliferation ability (all *P* < 0.05, Fig. 4b).

## Discussion

GDM is a healthy burden among pregnant women and can lead to poor pregnancy outcomes. HG as a characteristic of GDM, inducing the inhibited trophoblast cell proliferation and thereby resulting in the impaired development of placenta. This study investigated the expression of miR-132 in GDM patients and explored the regulatory effects of miR-132 on cell proliferation of trophoblast cell upon HG treatment. The data of this study shown that the expression of miR-132 in serum and placenta tissues was decreased in the GDM patients compared with the healthy pregnant women. The serum miR-132 expression was negatively correlated with the fasting blood glucose of the patients and had relative high diagnostic accuracy for the patients with GDM. HG induced the suppressed cell viability and reduced miR-132 expression in both HTR-8/SVneo and BeWo cells. By in vitro regulation, the inhibited trophoblast cell proliferation by HG was enhanced by the overexpression of miR-132.

Numerous miRNAs have been found to be abnormally expressed in various human diseases [18, 19]. These aberrant miRNAs are highlighted for their clinical applications and therapeutic potentials in the disease development and progression [20]. For example, serum expression of miR-30d has been reported to be downregulated in acute heart failure patients compared with the healthy controls, and the abnormal miR-30d was determined as a candidate diagnostic and prognostic biomarker for this acute disease [21]. Increased expression of miR-483-5p was detected in esophageal squamous cell carcinoma tissues, and its dysregulation was demonstrated to serve as an independent prognostic biomarker of this malignancy [22]. In type 2 diabetes mellitus patients, serum elevated miR-7 was proved to be a promising diagnostic biomarker and its expression could be used as a potential predictor of microvascular complications [23]. For the patients with GDM, some miRNAs with aberrant expression profiles have also been reported, such as miR-20a-5p [24], miR-137 [25] and miR-98 [26]. In the present study, we found the significantly decreased expression of miR-132 in the serum and placenta tissues in GDM patients compared with the healthy pregnant women. Moreover, a negative correlation was demonstrated between the serum miR-132 levels and the fasting blood glucose concentration in the patients with GDM. Thus, we believed that the aberrant miR-132 might also play an important role in the pathogenesis of GDM.

Serum examination for various biomarkers is an established well diagnostic strategy for human diseases, including GDM [27]. MiRNAs are stable in serum and have been described as a group of pivotal diagnostic candidates for numerous human diseases, such as malignancies [28], diabetes mellitus [29] and also GDM [24]. Given the decreased serum expression of miR-132 in GDM patients, a ROC curve based on miR-132 was plotted and revealed that the reduced miR-132 expression might be a potential diagnostic biomarker for GDM patients with relative high sensitivity and specificity. Serum aberrant expression of miR-132 has previously reported to serve as a candidate biomarker in some other diseases, including hepatocellular carcinoma [30] and mild cognitive impairment [31]. The miRNAs, including miR-132, are released by cells into different body fluids especially the blood, and exist stably with high resistance to harsh conditions and RNase digestion [32]. Thus, we believed that serum miR-132 may serve as a useful biomarker for human GDM.

As we all known that the regular growth and development of placenta is one of the most important step in a healthy pregnancy. However, the occurrence of GDM always along with the maldevelopment of placenta [6]. HG, as a characteristic of GDM, leads to the impaired function of trophoblast cells, thereby suppressing the normal development of placenta [33]. In the current study, the proliferation of trophoblast cell HTR-8/SVneo and BeWo was obviously inhibited by the HG treatment, which is consistent with the previous reports [33]. Additionally, HG treatment was also found to result in an inhibition in the expression of miR-132 in the trophoblast cells. Furthermore, the gain-of-function analysis indicated that the suppressed trophoblast cell proliferation induced by HG could be rescued by the overexpression of miR-132. miR-132 has been previously reported to regulate cell proliferation in pancreatic cancer [34] and laryngeal squamous cell carcinoma [35]. Thus, we considered that miR-132 might exert a protective role against GDM by enhancing the trophoblast cell proliferation. Zhang et al. has reported that miR-132 could facilitate cell proliferation of pancreatic cancer cells by targeting phosphatase and tensin homolog (PTEN) [34]. PETN is a major upstream inhibitor of the PI3K/AKT signaling, and has been identified as a critical molecule in GDM [36]. Li et al. found that plasma PTEN levels was associated with insulin resistance of GDM [37]. In addition, the increased expression of PTEN mediates the regulatory effects of miR-21 [38] and miR-144 [39] on the biological function of trophoblastic cells. Thus, we suspected that protective action of miR-132 in GDM might also achieved through targeting PTEN. The precise mechanisms underlying the role of miR-132 in GDM need to be investigated in future studies.

## Conclusion

Taken together, the data of this study demonstrated that the expression of miR-132 in serum and placenta tissues is reduced in the patients with GDM and serum miR-132 expression serves a promising non-invasive diagnostic biomarker for the screening of GDM cases. In addition, the upregulation of miR-132 can abrogate the inhibiting effects of HG on trophoblast cell proliferation, indicating that miR-132 may play a protective role in GDM through promoting trophoblast cell viability.

## Data Availability

All data generated during this study are included in this published article.

## Abbreviations

AUC: Area under the curve
CCK-8: Cell counting kit-8
DMEM: Dulbecco’s modified Eagle’s medium
FBS: Fetal bovine serum
GDM: Gestational diabetes mellitus
HG: High glucose
miR-132: microRNA-132
miRNAs: microRNAs
qRT-PCR: quantitative real-time PCR
ROC: Receiver operating characteristics curve
T2DM: Type 2 diabetes mellitus
